# Extensive Evolution of Cereal Ribosome-Inactivating Proteins Translates into Unique Structural Features, Activation Mechanisms, and Physiological Roles

**DOI:** 10.3390/toxins9040123

**Published:** 2017-03-29

**Authors:** Jeroen De Zaeytijd, Els J. M. Van Damme

**Affiliations:** Lab Biochemistry and Glycobiology, Department of Molecular Biotechnology, Ghent University, Coupure links 653, B-9000 Ghent, Belgium; Jeroen.DeZaeytijd@ugent.be

**Keywords:** b-32, cereals, JIP60, Ribosome-inactivating proteins, RIP

## Abstract

Ribosome-inactivating proteins (RIPs) are a class of cytotoxic enzymes that can depurinate rRNAs thereby inhibiting protein translation. Although these proteins have also been detected in bacteria, fungi, and even some insects, they are especially prevalent in the plant kingdom. This review focuses on the RIPs from cereals. Studies on the taxonomical distribution and evolution of plant RIPs suggest that cereal RIPs have evolved at an enhanced rate giving rise to a large and heterogeneous RIP gene family. Furthermore, several cereal RIP genes are characterized by a unique domain architecture and the lack of a signal peptide. This advanced evolution of cereal RIPs translates into distinct structures, activation mechanisms, and physiological roles. Several cereal RIPs are characterized by activation mechanisms that include the proteolytic removal of internal peptides from the N-glycosidase domain, a feature not documented for non-cereal RIPs. Besides their role in defense against pathogenic fungi or herbivorous insects, cereal RIPs are also involved in endogenous functions such as adaptation to abiotic stress, storage, induction of senescence, and reprogramming of the translational machinery. The unique properties of cereal RIPs are discussed in this review paper.

## 1. Introduction

Ribosome-inactivating proteins (RIPs) are enzymes that can irreversibly inhibit protein translation by depurinating the ribosomal RNA. Despite the fact that RIP genes are also found in fungi, bacteria, and even in some insects, they are thought to have originated in plants [1,2]. The history of RIP research was recently summarized by Bolognesi et al. [3]. Although the term “ribosome-inactivating proteins” was only introduced in 1982 [4] and the biological activities of these proteins were elucidated in 1987 [5] research on RIPs dates back to the nineteenth century when the toxicity of seeds from the plants *Ricinus communis* and *Abrus precatorius* was attributed to the presence of the proteins ricin and abrin, respectively. These two toxins were the first RIPs discovered and the scientific community was highly interested in their toxic properties. The proteins were first labeled as haemagglutinins because of their haemagglutination activity [6,7]. Olsnes and Pihl [8,9] discovered that both ricin and abrin consist of two polypeptides linked together by a disulfide bridge. The agglutination activity of the proteins was attributed to the *C*-terminal B-chain which corresponds to a galactose-specific lectin domain. Endo and Tsurugi [5] showed that the *N*-terminal A-chain of the proteins possesses an enzymatic activity which enables them to remove a specific adenine residue from the conserved sarcin/ricin loop (SRL) of the 28S rRNA, and consequently these proteins were referred to as rRNA N-glycosidases. Later it was reported that these proteins depurinated ribosomal RNA at multiple sites [10] and also removed adenine residues from other substrates such as herring sperm DNA, poly(A) and Tobacco Mosaic Virus (TMV) RNA [11]. For these reasons RIPs were considered as polynucleotide:adenosine glycosidases (PAG) [11,12,13]. Some authors also attributed other more exotic enzymatic activities to RIPs such as DNAse [14], RNAse [15], chitinase [16], phosphatase [17], lipase [18], and superoxide dismutase activities [19]. It cannot be excluded that these presumed activities were due to contaminants in the RIP preparations as reviewed in Peumans et al. [20]. However, more recent studies suggest that at least some RIPs from several *Phytolacca* species and *Beta vulgaris* possess endonuclease activity and/or superoxide dismutase activities. In these studies the necessary precautions were taken to exclude the possibility that the reported activities were due to contaminants [21,22,23,24,25,26]. Therefore the reported activities can be attributed to a particular structural configuration in these RIPs but cannot be considered a general feature of RIPs [22].

Following the elucidation of the enzymatic activity of ricin and abrin, RIPs were reported in a lot of plant species (reviewed by Schrot et al. [27]). Interestingly, some of these proteins did not possess a B-chain and consisted solely of the A-chain with RIP activity. This finding led to the classification of RIPs. Type-1 RIPs only consist of a RIP domain whereas the term type-2 RIPs refers to proteins composed of an enzymatic domain linked to a lectin domain.

Following the discovery of the cereal RIPs JIP60 (barley) and b-32 (maize) the term “type-3” was introduced by some authors [20,28,29] while others just considered these RIPs as atypical type-1 RIPs [30]. Alternatively, based on the domain architecture and evolutionary background type-1 and type-2 RIPs are called type-A RIPs and type-AB RIPs, respectively [2].

Type-A and type-AB RIPs differ in their cytotoxicity. In general, type-AB RIPs are toxic to animal cells since these RIPs can bind sugar moieties on the cell surface through their lectin domain, promoting their uptake in the cell. The absence of this glycoconjugate-binding activity explains the lower toxicity of RIPs devoid of a lectin domain. However, when type-A RIPs succeed to access the cytosol they also show cytotoxicity [31]. The existence of non-toxic type-AB RIPs emphasizes the fact that there are large differences in the toxicity of RIPs [32]. Overall, the toxicity is the result of the combined effects of: (i) Efficient binding to the cell surface; (ii) Uptake; (iii) Ability of the protein to reach the cytosol of the host cell; and (iv) Intracellular stability.

Because of their toxic effects some RIPs have a long history of being exploited for medicinal purposes. For example, trichosanthin (TCS) has long been used as a natural abortificient. The use of RIP producing plants in traditional medicine has recently been reviewed by Polito et al. [33]. Also in modern medicine there is a lot of interest in the toxic properties of RIPs. This interest increased after it was shown in 1970 that ricin and abrin were more toxic to tumor cells than to normal cells [34]. Ever since a lot of effort has been invested in exploring how to use RIPs to combat cancer. Initially some promising progress was made with immunotoxins where a RIP domain was coupled to an antibody, targeting the toxin specifically to tumorous cells (reviewed by de Virgilio et al. [35]). In some cases the RIP domain was not coupled to an antibody but to other targeting molecules, such as for example transferrin, since malignant cells overexpress the transferrin receptor [35]. Although some of these RIP based toxins showed promising results in clinical trials, a lot of problems arose concerning immunogenicity and aspecific toxicity of the toxins. The use of biomaterials such as gold nanoparticles, polymer/lipid nanoparticles, and nanocapsules allowed better control of the immunogenicity and delivery of these RIP based toxins as recently reviewed by Pizzo and Di Maro [36].

Several lines of evidence suggest that RIPs are involved in plant defense. First, the expression of several RIP genes is regulated by both abiotic and biotic stress factors. For example, the JIP60 gene from barley is only expressed in senescing leaves or leaves treated with methyl jasmonate (MeJA) [29,37]. Jiang et al. [38] showed that RIP genes from rice are stress responsive. Furthermore, overexpression of the OsRIP18 gene yielded rice plants that were more tolerant to salt and drought stress [39]. Second, feeding experiments with artificial diets as well as experiments with transgenic plants overexpressing RIPs showed the antiviral, antifungal, and insecticidal properties of RIPs as recently reviewed [40,41,42].

Though RIPs are present in a lot of different plant species, they are not ubiquitous, as evidenced by the absence of RIP genes from the *Arabidopsis* genome. Schrot et al. [27] recently provided an updated list of all known RIPs from plants. In the present review we will focus on RIPs from Poaceae, since this group of proteins differs in many aspects from RIPs of other plant species. Cereal RIPs have evolved at a higher rate resulting in some unique structures, domain architectures, activation mechanisms, subcellular localization, and physiological roles. To our knowledge only two other reviews have been published dealing with cereal RIPs in general [43,44]. Both reviews mainly focus on the b-32 RIP from maize. In view of the new data and insights generated in recent years an updated review on cereal RIPs can be justified.

## 2. Evolution and Unique Domain Architecture of Cereal RIPs

Initial studies on the evolution and taxonomical distribution of RIPs were merely based on the knowledge gathered from proteins that had been purified and characterized. Consequently only RIPs that are present in sufficient amounts to be purified are included in these phylogenetic analyses. Our research group performed some extensive in silico analyses based on both BLASTp and tBLASTn searches in genome as well as transcriptome databases. This allowed the mapping of the taxonomical distribution of RIPs and the elaboration of an evolutionary model for the RIP domain [1,2]. In 2013, Lapadula et al. [45] suggested a slightly different evolutionary model. The main difference between the two models is the explanation given for the occurrence of RIPs over different taxa like plants, bacteria, fungi, and some insects. While our research group is convinced that the presence of RIPs in non-plant taxa can be explained through horizontal gene transfer from the plant RIP lineage, Lapadula et al. [45] favor the idea that some paralogous genes exist in the common ancestor of bacteria and eukaryotes. The main weakness of this hypothesis is the absence of RIP genes in Archaea [45]. In the interest of this review we will only discuss the evolution of RIPs in the plant kingdom. The main findings from our in silico analyses indicate that: (i) RIPs are not ubiquitous since RIP sequences are absent from 24 out of 42 completed plant genomes (including *Arabidopsis*); (ii) The existing list of RIPs needs to be extended horizontally (more species) and vertically (more RIP genes per species); (iii) Extended RIP gene families are common in Poaceae/cereal species. The RIP genes from this family show some remarkable differences with other plants in terms of domain architecture of the RIP genes and complexity of the RIP gene family, suggesting a unique, quite recent, evolutionary process within the Poaceae/cereal species [2,46].

RIPs are classically divided into type-1 RIPs, that only consist of an A-chain with N-glycosidase activity, and type-2 RIPs in which the A-chain is linked to a *C*-terminal lectin B-chain. Special cases like the b-32 maize RIP (which can be considered a type-1 RIP that undergoes proteolytic activation) and the JIP60 from barley (where an *N*-terminal RIP-domain is fused to a *C*-terminal domain that shows high sequence similarity to the eukaryotic translation initiation factor 4E (eIF4E)) were classified as “type-3 RIPs”. New insights into the evolution of RIP genes and the complexity of several Poaceae/cereal RIPs in terms of domain architecture no longer fit this nomenclature and give impetus to readdress the classification system. Proteins/genes consisting of a single N-glycosidase domain will be referred to as the “A type” and the chimeric forms as the “AN type” whereby N designates the different (unknown) *C*-terminal domains. A^∆N^ RIPs are RIPs consisting only of an N-glycosidase domain resulting from the deletion of the N domain from the AN chimeric, whereas A^U^ RIPs have an unknown origin [2].

**Figure 1:**
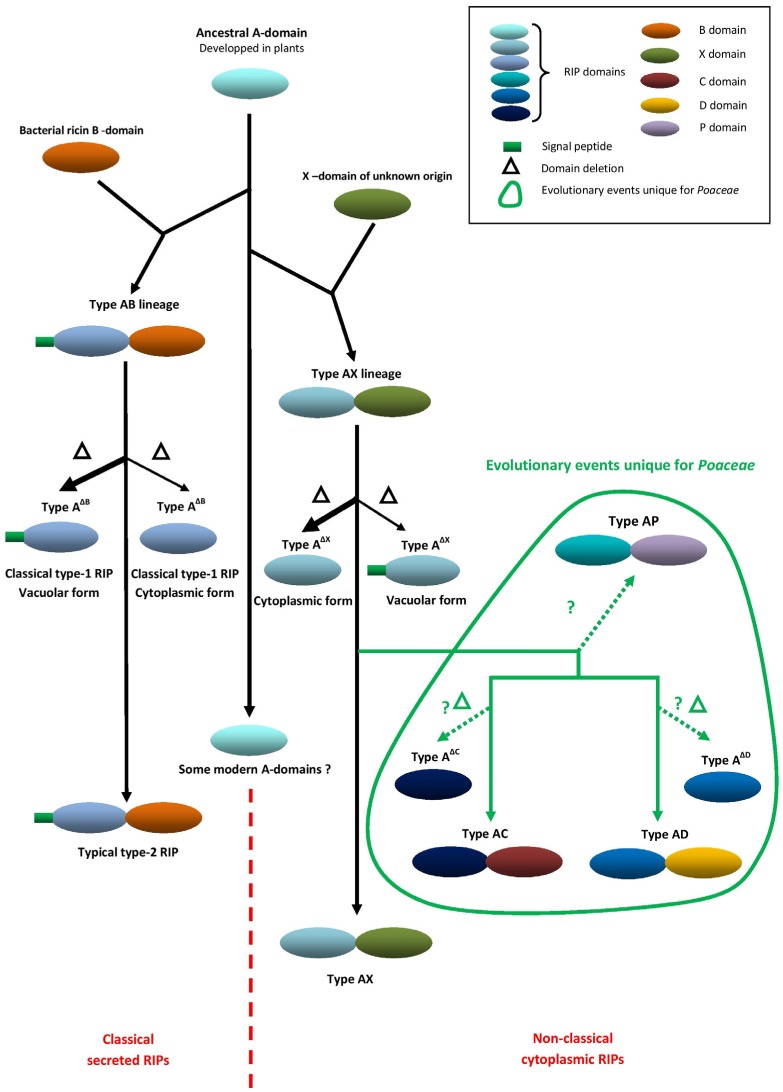
Evolutionary model for ribosome-inactivating protein (RIP) genes in plants.

According to the evolutionary model (Figure 1), an ancestral RIP domain was developed in plants. Next, it is hypothesized that a fusion with a bacterial B-chain (lectin) by horizontal gene transfer gave rise to the classical lineage of typical AB RIPs like ricin. This assumption is based on the widespread distribution of B-chain encoding sequences in all major prokaryotic and eukaryotic taxa [1,2]. Furthermore, sequence comparisons revealed that the B-chain of plant type AB RIPs shares a higher similarity with the carbohydrate-binding part of an β-glycosidase-like glycosyl hydrolase and an α-L-arabinofuranosidase B family protein from the Actinomycete bacterium *Catenulispora acidiphila* than it does with B-chains of other eukaryotes [1]. Domain deletions gave rise to the classical A^∆B^ RIPs. In addition to these typical RIP lineages a second lineage of chimeric plant RIPs, called the AX type, was established. This lineage is the result of a domain fusion with an X domain of unknown origin. Apart from the nature of the *C*-terminal domain, there is a very important difference between the AB and AX lineages. While classical AB RIPs are synthesized on the rough endoplasmic reticulum and are subsequently secreted or sequestered to the vacuole or cell wall, the RIPs descending from the AX lineage are synthesized without a signal peptide on free ribosomes, and hence remain in the nucleocytoplasmic space, where they can make contact with the host ribosomes. This difference in location between both AB and AX RIP lineages reflects a different function within plants and suggests an in planta activity of the AX RIPs in contrast to the generally accepted role of the AB RIPs in plant defense. A^∆B^ and A^∆X^ RIPs normally have the same subcellular localization as their parent chimeric forms although some exceptions occur. Based on the taxonomical distribution of AX and A^∆X^ RIPs, the ancestral AX gene is suggested to have originated before the separation of monocots and dicots. However, the exact timing of this event is hard to predict [2,46].

Next to the AB and AX forms, other chimeric RIP forms such as the AC, AD and AP forms were found in the genomes of some Poaceae/cereal species. The so-called “type-3 RIP”, JIP60 from barley, is probably the archetype of the AC group but these AC forms also occur in other cereal species. The AD type RIPs possess a *C*-terminal domain of yet another origin. In the AP forms an A domain is linked to a peptidase domain resulting in proteins with possible dual enzymatic activities. These forms are present in both *Oryza* and *Triticum*. Table 1 shows the occurrence of the different RIP forms in major cereal species [2,46].

The AC, AD, and AP forms presumably have the same subcellular localization as the AX forms. Low sequence identity exists between AC, AD, and AX forms suggesting that these forms evolved from an ancestral AX lineage. In the Poaceae family there are a lot of A-form RIP genes for which the origin cannot be traced. They are denominated as type-A^U^ RIPs. Possibly the AC and AD forms also gave rise to secondary A^∆C^ and A^∆D^ forms, though this assumption is rather hypothetical. An alternative hypothesis is that the A^U^ RIPs directly evolved from an ancestral A-form [2,46].

In summary, the RIP gene family evolved at an enhanced rate in Poaceae/cereal species. This assumption is supported by the large size and the heterogeneity of the RIP gene family within these species and the occurrence of chimeric forms that are not seen outside this clade. Because of the complexity it is very hard to reconstruct the exact evolution of the RIP gene family within the Poaceae/cereals. For example, in the *Oryza sativa* genome more than 30 genes with a RIP domain were identified [38]. Our preliminary screenings indicated that the RIP families in *Triticum aestivum* and *Hordeum vulgare* are of a similar complexity as in rice, whereas those of *Sorghum bicolor* and *Zea mays* are less complex. In future, more and better genomic and transcriptomic sequencing data can hopefully solve the complex evolutionary puzzle of the RIP gene family in Poaceae/cereal species. In parallel it is also very important to conduct biochemical analyses and to characterize the biological activity of these interesting RIPs. Together this will bring us closer to clarifying the physiological function of this unique cereal RIP family.

## 3. Biological Activity of Cereal RIP Domains

### 3.1. Structure of Non-Cereal and Cereal RIP Domains

#### 3.1.1. Structure and Active Site of Classical Non-Cereal RIPs

RIPs are N-glycosidases that can depurinate a specific adenine residue of the conserved SRL in the rRNA of prokaryotic or eukaryotic ribosomes. In order to fulfill their function, these proteins have evolved to near enzymatic perfection [47]. All RIPs are characterized by a typical three dimensional (3D) fold. Ricin, the RIP from *Ricinus communis*, was the first RIP for which the 3D structure was resolved at atomic level [48]. Ricin is also by far the best studied RIP and can be considered as a representative model for the RIP family. To date the 3D structures of more than 20 RIPs, both A and AB forms, have been resolved and studied in detail [49,50].

The structure of the A domain is highly conserved, irrespective of the domain architecture of the protein. While some authors suggest that the N-glycosidase domain consists of three domains [51], it is generally accepted that the A domain contains two subdomains [50,52,53]. The large *N*-terminal subdomain is composed of six α-helices and a six-stranded mixed β-sheet, and the small *C*-terminal subdomain consists of an anti-parallel β-sheet and an α-helix with a bend in the middle [49].

The specificity pocket of RIPs, responsible for binding of the substrate adenine of the SRL, is generally found in the cleft between the *N*-terminal and *C*-terminal subdomains [50]. The residues in the specificity pocket that are important for the RNA N-glycosidase activity are absolutely invariant (Table 2). The Tyr residues are involved in the stacking of the substrate adenine. Residue Y80 (in RTA) can adopt different conformations depending on the liganded state. For example, in the native ricin structure Y80 blocks the active site cleft and a ligand must displace the side chain to enable binding into the pocket [50]. Glu and Arg are the key residues for catalysis. Arg protonates the N3 of the ribose ring hereby forming a ribose carbocation which is stabilized by Glu [54]. Next to its stabilizing role, it is also proposed that Glu serves as a general base with nucleophilic water molecules for the depurination reaction [47,55]. The nucleophilic attack by the activated water molecule breaks up the N9-C1 glycosidic bond, releasing the adenine [35]. Many RIPs from dicotyledonous plants possess a second glutamate (Glu) residue in the active site that serves as a back-up when the catalytic Glu is mutated [47,56,57].

#### 3.1.2. Structure and Active Site of Cereal RIPs

Thus far, the crystal structures of two cereal RIPs have been determined, in particular the maize RIP1 and bRIP1 from barley [58,59]. Comparison of the overall structures of these cereal RIPs to RIPs from other plants revealed some differences. The α-helix B and β-strand 6 in the *N*-terminal subdomain are absent in the maize RIP1 and the anti-parallel β-strands 7 and 8 in the *C*-terminal subdomain are replaced by a short α-helix in maize RIP1 [58]. Unique for the maize RIP1 is the presence of an inactivation region (see below). The bRIP1 from barley possesses three unique 3_10_-helices G1/G2/G3 and an extra *C*-terminal α9-helix. In contrast, α-helix 2 and 2 β-strands present in the A domain of ricin (RTA) and other RIPs are absent in bRIP1 [59]. Especially the additional G2 helix and the *C*-terminal α9 helix are of importance and will be discussed in more detail below.

An important feature of cereal RIPs is that they do not seem to have a back-up glutamate like non-cereal RIPs. This observation was made for the structures of maize RIP1 and bRIP1 [58,59] but sequence comparison suggests that this phenomenon is also observed in other known RIPs of the family Poaceae, such as rice RIP (GenBank: BAB85659) and JIP60 (GenBank: AAB33361), and raises questions about the importance of the backup glutamate residue [58]. The active site residues for the toxic ricin A-chain (RTA), maize RIP1 and barley bRIP1 are identical (Table 2).

#### 3.1.3. Proteolytic Activation Mechanisms of Cereal RIPs

**Figure 2:**
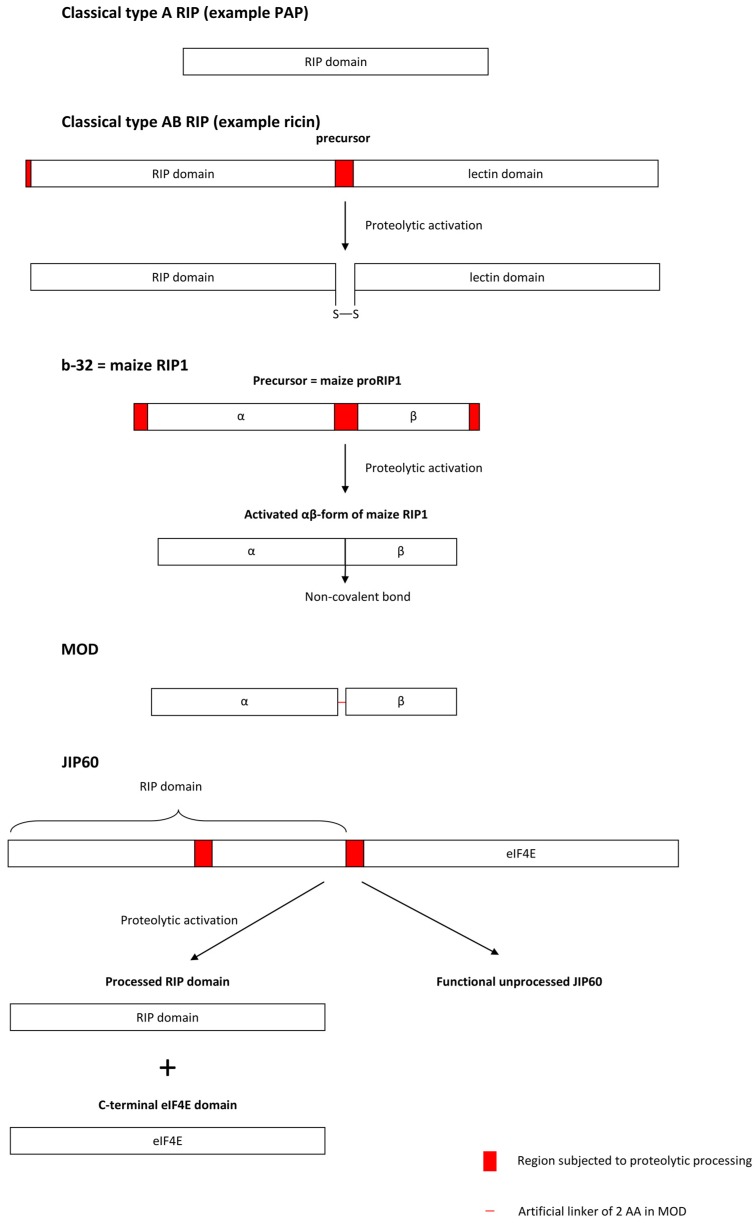
Activation mechanisms of the maize RIP1 (b-32) and the barley JIP60 in comparison with typical “Type-A” and “Type-AB” RIPs.

An important feature in the synthesis of cereal RIPs is the occurrence of unique activation mechanisms that have not been reported in RIPs from species outside the grass family. Walsh et al. [28] first reported that the maize RIP1 (pI of 6) is expressed and stored as an inactive precursor (proRIP1) in the endosperm. During germination it is proteolytically processed by endogenous proteases that remove 16 residues from the N-terminus, 25 residues from the central domain, and 12 residues from the C-terminus of the RIP rendering a two-chain active form (αβ-form) with a pI of 9. The two chains of 16.5 and 8.5 kDa in the active form are tightly associated but are not covalently linked (Figure 2). The Pro-RIP was previously described as the b-32 endosperm protein [60,61,62,63]. Although the processing of proRIP1 is achieved in vivo through the action of endogenous proteases, the activation can also be performed in vitro by a variety of nonspecific proteases such as papain and subtilisin Carlsberg. Purified proRIP1 is about 10,000 times less active than the processed αβ-form [28]. Activity assays with deletion mutants representing the different naturally occurring processing events allowed the contribution of different peptides in suppressing the activity of the maize RIP1 to be checked. These experiments clearly showed that the 25-amino acid internal insertion was the primary inactivating element of the pro-RIP. Removal of the *N*- or *C*-terminal sequence only increases the activity by 6- or 5-fold, respectively, whereas MOD, an active mutant of maize RIP1 in which the central internal inactivation region of proRIP1 is replaced by a 2-residue short linker, was reported to be about 650 times more active than proRIP1 in an in vitro ribosome inactivating activity assay based on a rabbit reticulocyte lysate [28,58,64]. However, proteolytically activated proRIP1 is still more active than the active MOD mutant [65]. The excision of an internal peptide to render an active form resembles the processing of certain human hormones, such as insulin, and represents a unique activation mechanism that is not described for other non-cereal RIPs [66]. Bass et al. [67] first postulated that the internal inactivation region might exert its function by disrupting the protein fold and affecting key residues in the active site [67], but this is unlikely since the inactive proRIP1 shows a well-structured fold and the inactivation region is located more than 15 Å apart from the active site cleft. Rather the inactivation region is proposed to disrupt the interaction with ribosomal proteins [68].

The maize b-32 RIP is not a standalone case. Bass et al. [67] reported a second maize RIP, referred to as RIP2, that is not predominantly located to the endosperm. Although gene expression for maize RIP2 is regulated in a significantly different manner, maize RIP2 showed a similar activation mechanism as reported for maize RIP1. ProRIP2 also contains an internal 19-amino acid peptide (in a similar region as in proRIP1) that is very rich in acidic residues, suggesting that this stretch of amino acids would also serve as an inactivation region and should be removed proteolytically for activation of the protein. Indeed, addition of the unprocessed form did not inhibit translation in a cell-free system based on a rabbit reticulocyte lysate. However, pretreatment of the proRIP2 with papain resulted in inhibition of translation. Since the proRIP2 is not specifically expressed in seed tissue as is the case with proRIP1, the question remains whether activation in vivo is caused by exposure to endogenous proteases (as shown for proRIP1 during germination) or by exposure to proteases introduced by invading pests or pathogens [67]. Chuang et al. [69] reported that proRIP2 can be processed in the midgut of fall armyworm into the active form.

The proteolytic processing of inactive RIP precursors into their active forms is not only restricted to maize. Hey et al. [64] analyzed seed extracts for different members of the subfamily Panicoideae by immunoblotting using antisera against the α and β fragments of the maize RIP1 [28]. The species *Z. mays parviglumis* (three accessions), Z. *luxurians,* Z. *mays mexicana,* *T. dactyloides,* and *Sorghum bicolor* were tested and immunoreactivity was observed for all extracts. However, a more distantly related species C*oix lachryma-jobi* showed no cross reactivity. Interestingly, in addition to the polypeptides of 32–34 kDa corresponding to the full length precursor protein the Western blot analyses also yielded polypeptides around 11–16 kDa, possibly corresponding to the separate α- and β fragments, suggesting proteolytic processing.

The proteolytic activation of RIP precursors can be extended beyond the Panicoideae subfamily since Chaudhry et al. [37] suggested that the JIP60 precursor from barley is processed in vivo. As described earlier, JIP60 is a 60 kDa jasmonate-inducible chimeric protein that consists of an *N*-terminal RIP domain and a *C*-terminal domain which displays similarity to eukaryotic translation initiation factor 4E [37,70]. Like maize RIP1, JIP60 differs from other RIPs in containing internal acidic peptides of 20–25 amino acids that will be removed during maturation of the protein. Western blot analyses confirmed that JIP60 was processed in extracts of jasmonate-treated leaves as well as after incubation with papain. It was suggested that at least two processing steps were necessary for rendering a fully active RIP. The *N*-terminal RIP domain should be separated from the *C*-terminal domain by proteolytic cleavage and an internal peptide in the RIP domain has to be removed, similar to the case of maize RIP1 (Figure 2). Full length JIP60 did not significantly inhibit protein translation in a system based on a reticulocyte lysate, whereas the *N*-terminal domain did inhibit translation to a certain extent. When the linker peptide in the *N*-terminal domain was removed, translation was inhibited almost completely [37].

In contrast, Reinbothe et al. [29] reported that unprocessed JIP60 did inhibit translation of plant mRNAs in rabbit reticulocyte lysates. However, they attributed this arrest of translation to the inhibition of the initiation step rather than the elongation step of translation as is the case with typical RIPs. Instead of acting as a typical RIP and blocking the elongation step, the unprocessed JIP60 caused the decay of the rabbit polysomes into their subunits, preventing the initiation step of translation. Unprocessed JIP60 also had this effect on barley ribosomes isolated from leaves exposed to MeJA for longer than 24 h, or from osmotically stressed or desiccated leaves. Polysomes from leaves treated with a water control were not cleaved by unprocessed JIP60.

Rustgi et al. [70] expressed artificial proteins in *E. coli* corresponding to the full length JIP60, the unprocessed *N*-terminal RIP domain and the processed *N*-terminal RIP domain. In a rabbit reticulocyte lysate, full length JIP60 and the processed RIP domain completely abolished translation, whereas the unprocessed RIP domain did not. A comparative analysis of the polysomal profiles allowed the mode of action of translation inhibition to be analyzed. In the case of unprocessed JIP60, there was a drastic decrease in ribosome binding to the transcripts which was not the case with the processed RIP-domain. These experiments confirmed the hypothesis of Reinbothe et al. [29] that unprocessed JIP60 can act as a ribosome dissociation factor. However, when proteolytically cleaved, the released processed *N*-terminal domain can act as a classical RIP as postulated by Chaudhry et al. [37]. It remains unclear why the latter research group did not observe any effect of unprocessed JIP60 on translation.

#### 3.1.4. “Switch Region” of Barley RIP

In 2012 the 3D structures of RIP1 from barley (bRIP1) in free form as well as in complex with adenine and AMP were determined [59]. In general the structure of bRIP1 shows high similarity with other RIP structures and most of the secondary structures are well superimposed. However, there are also some marked differences. The bRIP1 structure contains three unique 3_10_ helices G1, G2, and G3. Especially the G2 helix is of particular interest because this region displays two different conformations within the bRIP1 structure: it occurs as a 3_10_ helix or as a loop. As with other RIPs the geometry of Tyr84 (Tyr80 in RTA) in the active site can represent an “open” or a “closed” form depending on the liganded state. However, in bRIP1 the “open” or “closed” conformation of Tyr84 is correlated with a structural transition between a 3_10_ helix or a loop in this unique G2 region. When the G2 helix is formed, movement of a loop between the β-8 strand and the α-2 helix is blocked, thus preventing the transition from the open to the closed form needed for catalysis. On the contrary, when the region between the β-8 strand and the α-2 helix adopts a loop structure, the active site can close and enzyme catalysis can occur. Because of its putative role in the regulation of catalysis, this region was defined as a “switch region” [59].

Interestingly, this switch region is located at an equivalent position to the proteolytic cleavage site in the maize RIPs. These structural differences between apo and adenine-bound structures are not found in other RIP structures from dicotyledonous plants, which suggests a unique set of activation mechanisms in at least some cereal RIPs [59]. Since this region is also important for some RIPs (such as maize RIP1) for the interaction with ribosomes it might represent a major regulatory element of these proteins.

Barley bRIP1 is characterized by the presence of an additional *C*-terminal α-helix which is not found in maize RIP1 and RIPs from non-cereal species. However, several other cereal RIPs like those from barley, rice, and wheat have a strong sequence conservation in this *C*-terminal region [59]. At present it is unclear what the functional implications of this *C*-terminal α helix might be.

### 3.2. Interaction of RIPs with Ribosomes and Substrate Specificity

#### 3.2.1. Interaction of Classical Non-Cereal RIPs with Ribosomes

The substrate of RIPs for the depurination is the SRL of the rRNA. Depurination of naked rRNA is about 80,000 fold slower compared to depurination of whole ribosomes [71]. Though the SRL is highly conserved among all ribosomes, a lot of plant RIPs are able to depurinate eukaryotic ribosomes but not prokaryotic ribosomes. Therefore it was suggested that ribosomal proteins contribute to the susceptibility of rRNA to depurination by RIPs as well as to the differential sensitivity of ribosomes to RIPs [72,73,74]. Indeed, the fact that some RIPs such as TCS and ricin can only inactivate eukaryotic ribosomes while the RIP from *Phytolacca americana* called “pokeweed antiviral protein” (PAP), can inactivate both eukaryotic and prokaryotic ribosomes can be explained by the different interaction with ribosomal proteins. It was shown that PAP binds ribosomal protein L3 and that this interaction is required for depurination activity [72]. L3 is highly conserved between eukaryotic and prokaryotic ribosomes which explains the interaction of PAP with both types of ribosomes [75]. In contrast to PAP, a lot of other RIPs like TCS and ricin were shown to interact with the acidic ribosomal stalk proteins (P-proteins) [49,73,76]. The ribosomal stalk is part of the GTPase activation center [77] which plays a role in the binding of elongation factors [78,79,80]. Thus these RIPs might have evolved to hijack the translation-factor-recruiting function of the ribosomal stalk in reaching their target site on the rRNA [81]. Unlike L3, the ribosomal stalk proteins are not conserved between prokaryotes and eukaryotes. The stalk of bacterial ribosomes is formed by ribosomal protein L10 in complex with two or three copies of L12 homodimers [82,83] while the eukaryotic stalk is composed of a pentameric P-complex containing P0 and two P1/P2 heterodimers [84,85]. Prokaryotic ribosomes are insensitive to TCS, but when the stalk proteins were replaced by their eukaryotic counterparts, the hybrid ribosomes became susceptible to TCS. These data suggest that the difference in ribosomal stalk proteins explains why RIPs like TCS and RTA are not active against prokaryotic ribosomes compared to PAP.

TCS and RTA interact with the P2 peptide, a conserved *C*-terminal 11 residue-sequence “SDDDMGFGLFD” of the stalk proteins and this property was suggested to be a general theme for many RIPs [49,73,86]. The binding mechanism of TCS to the P2-peptide was first elucidated and revealed that the basic residues K173, R174 and K177 in TCS form favorable electrostatic interactions with the acidic DDD motif of the P2 peptide while a hydrophobic pocket accommodates the *C*-terminal LF residues of the P2 peptide [73,87]. Shi et al. [88] suggested a different but similar mode of interaction based on both electrostatic and hydrophobic interactions for RTA. Residues R189 and R193 of RTA bind the SDDD motif electrostatically and the LF motif is inserted into a hydrophobic pocket [88]. However, Fan et al. [89] reported that the DDDM motif contributes little to the interaction of P2 with RTA, and that the binding is the result of hydrophobic interactions and the formation of some hydrogen bonds between the F114 and D115 of P2 and R235 of RTA [89]. For both TCS and RTA hydrophobic forces dominate the interaction with the P proteins.

#### 3.2.2. Interaction of Cereal RIPs with Ribosomes

Maize RIP1 was also reported to interact with the P2 peptide of the acidic stalk proteins [58,68,90]. However, the interaction between maize RIP1 and the P2 peptide is significantly different compared to TCS and RTA. As mentioned previously, the β-strands 7 and 8 of the *C*-terminal domain that form the P2 binding site on TCS, are replaced by an α-helix in maize RIP. Therefore it was suggested that the P2 binding site in maize RIP1 might be different compared to other RIPs [58]. Using chemical-shift perturbation NMR (CSP-NMR) analysis the P2 binding site of maize RIP1 was mapped to a stretch of four lysine residues (K143–146) in the *N*-terminal part of the maize RIP1 [68]. Surprisingly this site is located next to the inactivation loop of maize proRIP1, reinforcing the hypothesis that the inactivation region is involved in sterically hindering ribosome binding rather than changing the conformation of the activity cleft. This assumption was confirmed by analyses whereby P2 was immobilized on a Sepharose column and subsequently challenged with both MOD and proRIP1. Only MOD could bind to P2 whereas proRIP1 could not. A MOD [K158A–K161A] mutant also did not bind to the column, confirming that binding is indeed established by the lysine stretch. Further pull down assays on the P2 column using buffers with different ionic strength proved that the interactions between maize RIP1 and P2 are electrostatic in nature, since binding of maize RIP1 was decreased with increasing salt concentrations. This was not the case for RTA or TCS. Furthermore removal of the FGLFD motif of the P2 peptide did not reduce interactions with maize RIP1 as it did for RTA and TCS [90]. Taken together these data suggest that maize RIP1 binds to P2, only after activation, and does so with a binding region that is very different from those of other RIPs. The binding is also solely dependent on electrostatic interactions and does not rely on hydrophobic interactions as classically seen with other RIPs [58,68,90]. Since maize RIP1 is the only cereal RIP thus far for which the interaction mechanism with ribosomal proteins was revealed, it is hard to predict if this unique mode of binding is valid for all cereal RIPs or this is a standalone case. Lapadula et al. [91] showed that the KKKK motif found in maize RIP1 is absent in RIP sequences from species like *Hordeum vulgare*, *Oryza sativa,* and *Triticum aestivum*. It was only present in some sequences from *Zea* species [91]. Since other cereal RIPs like JIP60 and barley RIP1 share some structural similarities with maize RIP1 in that they are also processed in a similar way to maize RIPs (as shown for JIP60 [37]) or contain a ‘switch region’ in the region corresponding to the inactivation loop in maize RIP1 (as is the case in barley RIP1) [59] it will be interesting to study the binding of these cereal RIPs with the ribosomal proteins.

#### 3.2.3. Substrate Specificity of Classical Non-Cereal RIPs

Although interaction with different ribosomal proteins can account for the fact that some RIPs can inhibit both prokaryotic and eukaryotic ribosomes (like PAP) while others cannot, this does not immediately explain the fact that a lot of RIPs inhibit ribosomes of different eukaryotic sources with variable efficiency. The “SDDDMGFGLFD” motif of P2, that was proven to be essential in binding of a lot of RIPs to ribosomes, is conserved among all eukaryotes. So if P2 interaction is the only mechanism required for binding to ribosomes, RIPs should be capable of inhibiting ribosomes of different eukaryotic sources with the same efficiency, including plant ribosomes. However, this is not the case. For example, ricin is most active against mammalian ribosomes but yeast ribosomes are less sensitive [92]. RTA is around 23,000 times less active on plant ribosomes compared to mammalian ribosomes [93]. Type-2 RIPs in general are several-thousand-fold less active on plant ribosomes than on mammalian ribosomes [93,94] while type-1 RIPs can efficiently act on plant ribosomes [95]. To circumvent the depurination of host ribosomes, plants protect themselves from the effect of their RIPs. Most RIPs are synthesized with *N*-terminal signal sequences that target them to the endomembrane system, away from the ribosomes [96]. Additional carboxy terminal extensions are found in several RIP sequences that subsequently target them to the vacuole and prevent these proteins from becoming active before they reach the storage organelles [97,98,99]. Other RIPs like PAP are secreted and stored in the cell wall [100].

#### 3.2.4. Substrate Specificity of Cytoplasmic RIPs from Cereals

Interestingly, RIPs from cereals seem to lack signal peptides, suggesting they should reside in the cytosol where they can make contact with the host ribosomes [28,37,101,102]. This raises some interesting questions concerning their mode of action. Are cereal ribosomes insensitive to these RIPs or do these RIPs exert their physiological role by inhibiting conspecific ribosomes? Different studies show that both options are possible. Both the pro-form as well as the activated form of the maize RIP1 are not very active on conspecific ribosomes [64]. This also suggests that the removal of the inactivation loop is not a measure to protect the host ribosomes. No results are available for the effect of the second maize RIP [67] on conspecific ribosomes.

Tritin, the RIP found in wheat germ, was reported to be inactive against wheat ribosomes [103,104], while other studies suggested it could inhibit conspecific ribosomes at least to some extent [102,105]. Massiah and Hartley [104] reported the existence of a second RIP in wheat that was not found in the seeds but in the leaves. Therefore they referred to the originally discovered tritin as tritin-S while the leaf form was given the name tritin-L. The authors showed that the S-form was inactive against conspecific ribosomes while the L-form could depurinate wheat ribosomes. Furthermore they reported that the S-form needed ATP as a cofactor in order to be enzymatically active while this does not hold true for the L-form. Sawasaki et al. [106] showed that tritin mediated the programmed senescence of the wheat coleoptiles by cleaving conspecific ribosomes. Although the authors did not mention explicitly which tritin form they focused on, our investigation of the gene sequence revealed that it encodes the tritin-S form. Hence this result contradicts the findings from Massiah and Hartley [104].

While barley seed RIPs are not active against barley ribosomes [101], the JIP60 found in barley leaves is a special interesting case. It was mentioned earlier that full length JIP60 can act as a ribosome dissociation factor [29,70]. However, only ribosomes isolated from leaves that had been exposed to MeJA for more than 24 h, or ribosomes from osmotically stressed or desiccated leaves were susceptible to the unprocessed JIP60. When processed, the *N*-terminal RIP domain of JIP60 can act as a genuine RIP [37,70]. Dunaeva et al. [107] reported N-glycosidase activity of (processed) JIP60 in tobacco and barley, suggesting that also the processed form is active on plant ribosomes.

In general, the interaction with ribosomal proteins seems to play a very important role in determining the substrate specificity of RIPs. However, there can be important differences depending on the RIP under study. PAP, RTA, TCS and maize RIP1 all show different modes of interaction with ribosomal proteins. More studies should be conducted to elucidate the binding of RIPs to ribosomal proteins as well as their substrate specificity. This is especially the case for the interaction between plant ribosomes and cereal RIPs given their unique structure, activation mechanisms, and presumed cytosolic localization in the plant cell.

## 4. Physiological Roles of Cereal RIPs

RIPs are supposed to help plants in safeguarding them from pathogenic fungi, viruses, or insects. These classical defense functions were also attributed to cereal RIPs. However, there is evidence that several cereal RIPs also mediate some very unique in planta functions. A summary of the physiological roles reported for cereal RIPs is given in Table 3.

### 4.1. Role in Defense

#### 4.1.1. Maize RIP1

Maize RIP1, also called RIP b-32, is by far the most intensively studied cereal RIP. It is exclusively found in the endosperm of maize kernels. The expression of the RIP1 gene is controlled by the Opaque-2 (O2) locus, which also controls the expression of other typical endosperm proteins such as the 22 kDa zeins [108]. The first indication that the b-32 protein is involved in protecting the maize seeds from biotic stress factors came from the observation that *o2* mutants were more susceptible to fungal attack [109] and insect feeding [110]. Since then, a lot of research has been conducted to clarify the role of maize RIP1 in plant defense.

The proteolytic activation mechanism of maize RIP1 has already been discussed in this review. The proRIP1 showed an inhibitory effect on the growth of *Rhizoctonia solani* in vitro [111]. However, other authors reported that proRIP1 had no effect on *Aspergillus flavus* and *Aspergillus nidulans*, while the proteolytically activated RIP1 reduced the growth of the pathogens in vitro. Furthermore when the RIP1 protein was mutated to abolish the RIP activity the protein did no longer inhibit the fungal pathogens, suggesting that RIP activity is essential for the antifungal activity [112]. It is not clear why activation of the proRIP1 seems to be necessary for antifungal activity in some experiments while this is not a prerequisite in other assays. One explanation could be that in those cases where the pro-form was active against fungi, the protein was actually activated by proteases secreted by the fungus.

Next to these in vitro bioassays, a lot of experiments with transgenic plants expressing the maize RIP1 were conducted in order to check the antifungal effect of the protein in planta. Transgenic rice plants expressing the maize RIP1 did not significantly reduce the disease severity caused by the fungal pathogens *Rhizoctonia solani* and *Magnaporthe griseae* [113]. However, when the protein was overexpressed in tobacco RIP1 it did confer increased protection against *Rhizoctonia solani* [111]. Similarly, transformed wheat plants expressing maize RIP1 were more resistant to infection by *Fusarium culmorum*, the causal agent of *Fusarium* head blight. When immature spikes were inoculated with spores, the number of infected spikelets per head at seven and 14 days after inoculation was significantly lower in transgenic plants compared to control plants [114]. Leaf tissue colonization bio-assays conducted with *Fusarium verticillioides* and leaves from transgenic maize ectopically overexpressing the b-32 protein clearly showed a reduction in mycelial growth compared to wild type leaves. Furthermore the level of resistance was correlated to the RIP1 content in the transgenic leaves [115]. The antifungal properties of maize RIP1 have been reviewed in detail by Motto and Lupotto [43] and Lanzanova et al. [116].

Besides antifungal properties, the maize RIP1 also acts against predatory insects. Dowd et al. [117] tested the effect of purified proRIP1 as well as papain activated RIP1 on a wide array of insects. Five different species of caterpillars were used for feeding assays and five different beetle species were used in choice assays. The caterpillars used in the feeding assays were: corn earworms (*Helicoverpa zea*), fall armyworms (*Spodoptera frugiperda*), European corn borers (*Ostrinia nubilalis*), cabbage loopers (*Trichoplusia ni*), and Indian meal moths (*Plodia interpunctella*). Only the papain activated RIP1 showed some significant effect on the caterpillars and the degree of toxicity was strongly dependent on the species tested. The toxic effects ranged from 70% mortality for the cabbage looper to no effect on the Indian meal moth. For the choice assays the following beetle species were used: freeman sap beetles (*Carpophilus freemani*), strawberry sap beetles (*Stelidota geminata*), maize weevils (*Sitophilus zeamais*), and dusky sap beetles (*Carpophilus lugubris*). In contrast to the effects on caterpillars, both proRIP1 and activated RIP1 significantly deterred feeding in choice assays with relative feeding rates being reduced up to 6-fold.

Efforts were made to confirm these effects in planta by overexpressing the maize RIP1 in plants and subsequently checking the effects on herbivorous insects. Tobacco plants expressing an activated form of the maize RIP1 showed enhanced resistance against the corn earworm (*Helicoverpa zea*). Insect feeding was reduced on transgenic plants compared to wild type plants and there was a higher mortality in the population fed on the transgenic plants. The survivors were characterized by a reduced bodyweight. The degree of damage to the plants caused by *Helicoverpa zea* was significantly though inversely correlated with RIP1 levels present in the plants [118]. Tobacco hybrids, obtained by crossing a maize RIP1 expressing line with a tobacco line overexpressing the tobacco anionic peroxidase also showed enhanced resistance against the corn earworm (*Helicoverpa zea*) and the cigarette beetle (*Lasioderma serricorne*). Transgenic plants were less prone to feeding by both insects compared to wild type plants. This effect was again inversely correlated with overexpression levels of both proteins in the tobacco plants [119]. Finally, maize plants ectopically overexpressing both maize RIP1 and a wheat germ agglutinin were more resistant to feeding by larvae of the fall armyworm *(Spodoptera frugiperda*) and of the corn earworm (*Helicoverpa zea*), and this level of resistance was correlated with the levels of maize RIP1 and the agglutinin [120]. Together, these data suggest that the maize RIP1 plays an important role in protecting developing maize seeds against attacks by fungal pathogens and herbivorous insects.

#### 4.1.2. Maize RIP2

Compared to the maize RIP1, much less attention has been given to the second RIP identified from maize. As mentioned previously, similar to maize RIP1 the maize RIP2 is characterized by a proteolytic activation mechanism [67]. However, some important differences exist between the two proteins. The amino acid sequences of maize RIP1 and maize RIP2 share only 73% sequence similarity [121]. Furthermore the expression of RIP1 and RIP2 is differently regulated. While maize RIP1 is exclusively expressed in the kernel, maize RIP2 is expressed throughout the whole plant, except the kernel [67]. The RIP1 gene is found on chromosome 8 where it is under control of the opaque-2 regulatory locus and its expression is controlled developmentally. In contrast the RIP2 gene is located on chromosome 7 and RIP2 expression is responsive to environmental stimuli. Bass et al. [67] showed that RIP2 expression is higher upon drought stress, and a Chuang et al. [69] reported that RIP2 accumulates in maize leaves after caterpillar attack, suggesting a role for RIP2 in the defense against herbivory. This role of RIP2 was already hinted by the fact that the position of the RIP2 gene on chromosome 7 coincides with a strong QTL for caterpillar resistance [122]. Chuang et al. [69] reported that RIP2 expression strongly increased in leaves after feeding of the fall armyworm (*Spodoptera frugiperda*). Transcript levels for RIP2 as well as protein levels increased one hour after caterpillar attack and remained high during the 24 h monitoring period. Four days after initial feeding, RIP2 levels were still high in maize leaves. In contrast to caterpillar feeding, mechanical wounding did not alter RIP2 expression. Since several phytohormones like salicylic acid (SA), MeJA, and ethylene (ET) are known to be involved in the regulation of plant responses to biotic stress, the authors also investigated whether treatment with these hormones could trigger RIP2 expression. Only MeJA and ET induced RIP2 expression albeit only when combined with mechanical wounding. Treatment with ABA, a phytohormone involved in abiotic stress response, combined with mechanical wounding also induced RIP2 expression confirming the results of Bass et al. [67].

A second line of evidence for the role of RIP2 in herbivory defense came from the fact that the RIP2 protein survived digestion in the midgut of the fall armyworm. Defense proteins are often resistant to the proteases found in the midgut of caterpillars and can consequently be traced in the frass, while proteins not involved in defense mechanisms are easily degraded by the insect and are therefore not found in the frass. One of the predominant proteins found in the frass of fall armyworms fed on maize leaves was the maize RIP2. Furthermore, in the Western blot analyses polypeptides corresponding to the processed form of RIP2 were detected, suggesting that the protein is activated by insect proteases [69].

To confirm the presumed defensive role of the maize RIP2 against herbivorous insects, recombinant RIP2 was made and fed to larvae of fall armyworm. The concentration of recombinant protein in the artificial diet represented the amounts of RIP2 found in maize leaves under caterpillar attack. Both papain activated RIP2 as well as the unprocessed proRIP2 reduced the larval weight by 26% compared to larvae fed on BSA or buffer control. Immunoblotting of the frass of the fed larvae confirmed that proRIP2 was proteolytically activated in the midgut of the fall armyworm [69].

These data suggest that RIP2 is important in the inducible protection of the vegetative tissues of maize plants against fungal pathogens and insects, while RIP1 protects the developing seeds of the plant in a more constitutive manner.

#### 4.1.3. Sorghum RIP

A protein similar to the maize RIP1 was also detected in sorghum seeds. Antibodies raised against the maize RIP1 cross reacted with this sorghum RIP and the occurrence of multiple reactive polypeptides suggested proteolytic processing of the sorghum RIP, as observed with the maize RIP1 [64]. The sorghum RIP is a member of the group of antifungal proteins (AFPs) found in sorghum caryopsis. Other AFPs include sormatin, chitinase, and glucanase. The presence of these AFPs is also characteristic for caryopsis of other cereals such as barley, maize, and wheat [63,123,124,125]. Seetharaman et al. [126] extracted and partially purified these sorghum AFPs and tested their biological activity against the grain molding pathogens *Fusarium moniliforme*, *Curvularia lunata,* and *Aspergillus flavus* in vitro. They reported that a mixture containing several AFPs was most inhibitory against the pathogens. RIP levels in sorghum caryopsis are highest at 15 days post anthesis and subsequently decreased, while the other AFP levels increase during development and only go down when grain maturity is reached [127]. Rodriguez-Herrera et al. [128] compared the levels of these AFPs in grain mold resistant (GMR) and susceptible (GMS) sorghum lines and noticed that in environments with grain mold incidence, the levels of RIP, sormatin, and chitinase were higher in GMR lines than in GMS lines. In a grain mold free environment the RIP levels were also higher in the GMR group. In the GMR lines, the levels of RIP, sormatin, and chitinase were higher in environments with grain mold incidence compared to environments without grain mold. These data suggest that GMR lines express more AFPs under grain mold pressure than GMS lines. In addition, RIP levels seem to be higher in GMR lines regardless of the presence of grain mold [128]. The sorghum RIP thus seems to play an important role in protecting the seeds against fungal attack.

#### 4.1.4. Barley RIPs

Barley seeds contain three type-A RIPs, the most famous one being RIP30. The RIPs are very similar in amino acid sequence and can be considered isoforms [129]. Similar to the sorghum RIP, RIP30 is one of the antifungal proteins identified in barley seeds. Leah et al. [101] showed that RIP30 accumulates to high levels during late seed development, and inhibits the growth of the fungal pathogens *T. reesei* and *F. sporotrichioides* in vitro. However, the inhibitory effect was much more pronounced when a mixture containing the RIP and other AFPs such as glucanase and chitinase was used, suggesting that the effect of RIP30 is enhanced when the fungal cell walls are permeabilized by the action of these hydrolases [101]. Several experiments have been conducted to confirm the in planta antifungal effects of the barley RIP by overexpressing the protein in different plant species and subsequently examining the plant’s resistance against fungal pathogens. In a study from Oldach et al. [130] transgenic wheat overexpressing the barley RIP did not significantly reduce formation of powdery mildew (*Erisyphe graminis*) or leaf rust (*Puccinia recondita*) colonies while wheat plants overexpressing chitinase or an antifungal protein from the fungus *Aspergillus giganteus* did reduce the disease symptoms. Transgenic wheat plants overexpressing an apoplastic targeted version of the barley seed RIP were not or only moderately protected against *Erysiphe graminis* but did show protection against the powdery mildew (*Blumeria graminis*). Co-expression of other AFPs such as barley seed chitinase and barnase did not significantly improve this level of protection [131,132]. More recent studies have proven that the overexpression of a combination of RIP30 and chitinase is successful. Transformed Indian mustard (*Brassica juncea*) expressing both barley RIP and chitinase showed 44% reduction in *Alternaria brassicae* hyphal growth in an in vitro assay and transgenic plants sprayed with an *A. brassicae* spore suspension showed reduced numbers, sizes, and expansion of lesions compared to wild type plants [133]. Similarly, potato plants overexpressing a combination of RIP and chitinase showed enhanced resistance to *Rhizoctonia solani* in a greenhouse assay [134]. Chopra and Saini [135] reported that transformed blackgram (*Vigna mungo*) plants expressing the same set of genes arrested the growth of *Corynespora cassiicola* in an in vitro antifungal assay and the plants also showed less disease symptoms compared to wild type plants after being sprayed with a spore suspension.

## 5. In planta Functions

A lot of cereal RIP genes are characterized by the absence of a signal peptide and therefore will most probably reside in the cytoplasm where they can make contact with the host ribosomes. This interaction can have implications regarding the possible endogenous functions of these RIPs.

### 5.1. Storage Function of Cereal Seed RIPs

Next to being used as antifungal agents, the maize RIP1 and barley seed RIPs are also thought to fulfill a nutritional role. It was previously mentioned that the expression of the maize RIP1 is controlled by the opaque-2 locus, which is known to regulate the expression of the zeins. It was also noticed that the maize RIP1 is high in lysine and methionine, amino acids that are underrepresented in the zeins [136]. Therefore the maize RIP1 could serve as a storage albumin that compensates for the lower methionine and lysine contents in the major seed storage proteins [43,137]. A similar nutritional function was suggested for the barley RIP30 isoforms that are expressed specifically in the starchy endosperm. The starchy endosperm cells differentiate during development and at maturity they are metabolically senesced. Leah et al. [101] hypothesized that the barley seed RIPs could play a role in this process.

### 5.2. Rice RIPs

A genome wide survey of the RIP family in *Oryza sativa* spp*. Japonica* conducted by Jiang et al. [38] identified 31 genes encoding proteins containing a RIP domain. All these putative RIPs were named type-1 RIPs. qPCR analyses showed that these RIP genes are differentially expressed in leaves, roots, and panicles and that the expression of several genes was stress responsive [38].

Analyses conducted by our group identified slightly different numbers of rice RIP genes in the genomes of *Oryza sativa* [2,46]. In silico analyses of expression data from RNAseq studies confirmed the differential expression of the RIP genes in different tissues and under different abiotic stresses. While Jiang et al. [38] only identified type-A RIPs, we believe that some of these RIPs have an AN type architecture. Both RIP sequences with and without signal peptide were found. The fact that some RIPs are differentially expressed after the application of abiotic stress suggests a role in the abiotic stress response.

The gene RA39 reported by Ding et al. [138] is a tapetum specific gene that is highly expressed in rice tapetal cells at the meiosis stage. RA39 encodes a secreted RIP and the recombinantly produced protein exhibited RNA N-glycosidase activity. During microspore development tapetal cells have a nutritional function and are degraded in order to free up nutrients for the maturing microspores. This tapetum degradation can be seen as a type of programmed cell death. Since the timing of this process coincides with the spatio-temporal regulation of RA39 expression, the authors hypothesized that the RIP might play a role in programmed cell death [138].

The RA39 studied by Ding et al. [138] is referred to as the OsRIP18 by Jiang et al. [38]. Expression of an OsRIP18 promoter-GUS construct confirmed the tapetum specific expression. Furthermore Jiang et al. [38] showed that the tapetal expression increased after polyethylene glycol and salinity stress, suggesting a role of the gene in drought and salinity tolerance. Rice plants ectopically overexpressing the RIP showed significantly increased tolerance to drought and high salinity stress, substantiating the possible function of OsRIP18 in abiotic stress tolerance [39]. Micro-analysis revealed that 128 genes were upregulated in rice plants overexpressing the RIP gene compared to wild type plants. Five of these genes are also upregulated in wild-type plants stressed by drought or salinity compared to non-stressed wild-type plants. The authors concluded that OsRIP18 might exert its function by re-organizing the translational machinery.

### 5.3. Maize RIP2

As mentioned earlier, the expression of maize RIP1 and RIP2 is differently regulated. Chuang et al. [69] showed that next to caterpillar feeding, a combination of mechanical wounding and ABA triggered maize RIP2 expression. Furthermore Bass et al. [67] reported that the level of RIP2 transcripts as well as the protein levels increased when plants were subjected to drought stress. These data suggest that besides protecting maize leaves against insect herbivory, maize RIP2 can also be involved in the abiotic stress response.

### 5.4. Tritin

Although there are some contradictions in the literature concerning the different isoforms of tritin found in wheat and their respective action on conspecific ribosomes [102,103,104,105], it is safe to say that at least one isoform of tritin serves an endogenous role in wheat by acting on wheat ribosomes. Sawasaki et al. [106] showed that tritin together with an RNA apurinic site-specific lyase (RALyase) mediates coleoptile senescence by cleaving the sarcin/ricin domain of conspecific ribosomes. RALyase is an enzyme that cleaves the phosphodiester bond at the depurination site of tritin. It was suggested that this enzyme completes the translational arrest caused by tritin, by reducing the residual translational elongation of the depurinated ribosomes [139]. Senescence is an important developmental process which allows the plant to redistribute nutrients from sinks to developing plant parts [140]. Programmed cell death is an active process during senescence [141]. Sawasaki et al. [106] noted that senescence of wheat coleoptiles was accompanied by morphological changes such as discoloration and withering. These changes were most obvious starting from 12 days after sowing of the wheat seeds. Interestingly, RT-PCR analyses showed that transcript levels for tritin emerged in wheat coleoptiles starting from day 10 and the expression dramatically increased from day 11. RALyase on the other hand apparently is constitutively expressed during senescence. Furthermore starting from day 10, the wheat ribosomes also appeared to be damaged by the combined effect of tritin and RALyase. The correlation between the expression pattern of tritin and RALyase, the onset of morphological changes and the ribosomal damage associated with tritin and RALyase activity, strongly suggest an important regulatory role for tritin in wheat coleoptile senescence. This hypothesis was confirmed by creating transgenic tobacco plants expressing tritin or RALyase under the control of the glucocorticoid-inducible promoter. When RIP expression was induced in seedlings, the plants started to show a senescent phenotype. However, this was not the case in seedlings where RALyase expression was induced, indicating that tritin is the driving force behind the regulation of senescence [106].

### 5.5. JIP60

The JIP60 protein has already been discussed in terms of its domain architecture and proteolytic processing. Similar to classical RIPs the processed *N*-terminal RIP domain inhibits translation by blocking the elongation step [37,70]. The N-glycosidase activity of the RIP domain was proven in vitro using rabbit reticulocyte ribosomes, as well as in planta [107]. It was shown that the unprocessed JIP60 can also abolish translation of both plant and rabbit ribosomes though this process occurs in a completely different way as with the *N*-terminal RIP part. The full length JIP60 acts as a ribosome dissociation factor and blocks the initiation of translation rather than the elongation step, by splitting the polysomes in their subunits. Remarkably, JIP60 only had this effect on ribosomes from leaves that had been exposed to MeJA or from osmotically stressed or desiccated leaves [29,70]. While these features were already discussed above, we did not yet clarify the role of the *C*-terminal eIF4E domain and how the processing contribute to the in planta function of JIP60.

Rustgi et al. [70] performed in vitro translation assays where transcripts of different genes were used as a template. These transcripts represented both housekeeping genes and genes involved in photosynthesis (RBSC, actin, LHCB2) as well as other jasmonate inducible genes (JIP23 and thionin). Derivatives of JIP60 were added to the reactions to study their effect on the translation of the different transcripts. It has already been mentioned that both the complete JIP60 protein as well as the processed RIP domain completely abolished translation, albeit in a different way. Interestingly the effect of the eIF4E domain derivative had no effect on the translation of the transcripts of housekeeping genes or genes involved in photosynthesis but an increased translation of the transcripts from jasmonate inducible genes was detected. The authors also observed that these JIP transcripts were increased in the polysome fraction. These findings suggest that the eIF4E domain resulting from the processing of the JIP60 selectively promotes the translation of other JIPs [70].

**Figure 3:**
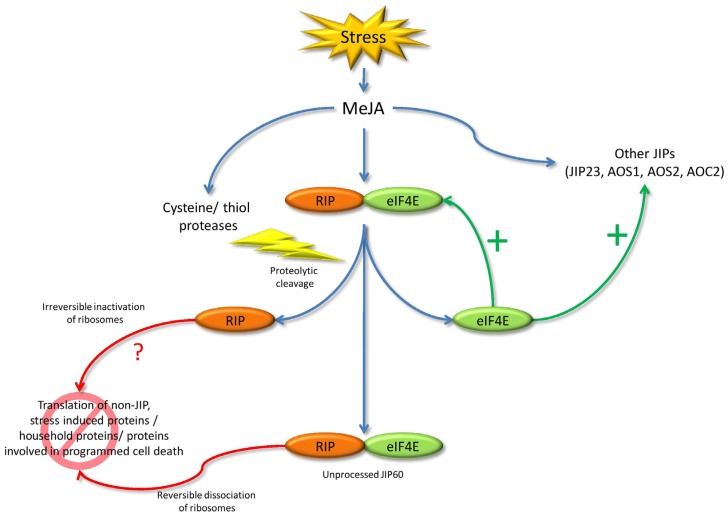
Working mechanism for JIP60 when the plant is subjected to stress (adapted from [70]). MeJA treatment affects the expression of several jasmonate-inducible proteins (JIPs) and proteases. Among the JIPs are JIP60, JIP23, and the enzymes involved in jasmonate biosynthesis:allene oxide synthases 1 and 2 (AOS1, AOS2) and allene oxide cyclase (AOC). JIP60 promotes the translation of JIPs through the action of the separate *C*-terminal domain, while unprocessed JIP60 as well as the separate *N*-terminal domain inhibit the translation of other proteins.

JIP60 is involved in the barley stress response. This is evidenced by the fact that its expression is dependent on MeJA, a phytohormone involved in plant defense. Rustgi et al. [70] also reported that the JIP60 gene is in close proximity to several QTLs for both biotic and abiotic stress resistance, confirming a probable role in stress response. JIP60 probably exerts its function by reprogramming the translational machinery in order to cope with unfavorable situations. Figure 3 represents a possible working model for JIP60.

## 6. Concluding Remarks

In this review we discussed the characteristics of cereal RIPs which distinguish them from their counterparts in other plant species. These unique features of cereal RIPs probably result from the extensive evolution of cereal RIP genes. Several cereal RIPs are known for their proteolytic activation mechanisms. Famous examples are the maize RIP1 and the JIP60 from barley but evidence exists that homologs also occur in other species such as sorghum. Although the bRIP1 from barley does not contain an inactivation region that needs to be removed, a “switch region” was reported in bRIP1 at a location corresponding to the location of the inactivation loop of maize RIP1. This finding argues that structural regulation of RNA N-glycosidase activity might be a more general phenomenon for cereal RIPs than previously thought. As seen with the maize RIP1, this structural regulation might be achieved by affecting the interaction with ribosomal proteins rather than changing the active site conformation.

Another important difference between cereal RIPs and classical plant RIPs is the lack of signal peptides and consequently their presumed cytoplasmic localization. This has some important implications for the possible in planta function of cereal RIPs. There are some contradictions in the literature with regard to the effect of cereal RIPs on conspecific ribosomes. While certain RIPs were reported not to act on the host ribosomes, others like tritin clearly exert their function by inhibiting cereal ribosomes. Even more, JIP60 only dissociates barley ribosomes in certain stress situations suggesting that barley ribosomes are somehow ‘marked’ for cleavage in specific situations. This underlines the potential complexity of the interaction between cereal RIPs and (conspecific) ribosomes.

Studies on the physiological roles of cereal RIPs often focus on one specific action of an RIP. For example, the role of maize RIP2 in the defense against caterpillars was investigated. However, although the maize RIP2 gene was reported to be upregulated by drought stress, its possible role in drought tolerance has not yet been clarified. Possibly cereal RIPs, and especially the leaf forms, can have a dual function. On the one hand they can function as classical defense proteins protecting the plant against herbivorous insects or pathogenic fungi, while on the other hand they can be involved in regulating endogenous processes such as the abiotic stress response or developmental processes like senescence. Future research on cereal RIPs should focus on both aspects and can gain more insight into the importance of this unique class of proteins found in cereals. A better understanding of the physiological importance of RIPs can lead to future exploitation of their properties to create more resilient crops. Next to being useful for agricultural purposes, the unique properties of cereal RIPs could also be exploited in other research fields. An example is the use of maize RIP1 in the combat against human immunodeficiency virus (HIV). The unique activation mechanism of maize RIP1 has been exploited to enhance the specificity of the RIP against HIV infected cells by the introduction of HIV-1 protease recognition sequences in the internal inactivation region [142].

## Figures and Tables

**Table 1:** Different ribosome-inactivating protein (RIP) forms in cereal species.

Species	RIP Gene Architectures Reported
*Oryza sativa* (rice)	A^u^, AC, AP
*Avena barbata* (oat)	A^u^, AC
*Hordeum vulgare* (barley)	A^u^, AC
*Triticum aestivum* (wheat)	A^u^, AB, AP
*Sorghum bicolor* (sorghum)	A^u^, AB
*Zea mays* (maize)	A^u^, AB, AC, AD

**Table 2:** Comparison of the active site residues of RTA, maize RIP1 and barley bRIP1.

RIP	Active Site Residues				
RTA	Y80	Y123	E177	R180	W211
Maize RIP1 = b-32	Y94	Y130	E207	R210	W241
Barley bRIP1	Y87	Y118	E175	R178	W213

**Table 3:** Summary of different physiological roles reported for cereal RIPs.

Species	RIP	Tissue	Role in Defense	In Planta Function
*Zea mays*	Maize RIP1 (= b-32)	Seeds	Antifungal, insecticidal	Storage function in seeds?
	Maize RIP2	Whole plant, except kernel	Expression upon herbivore attack, active against *Spodoptera frugiperda*	Involved in drought response?
*Sorghum bicolor*	Sorghum RIP	Seeds	Antifungal protein	Not reported
*Oryza sativa*	OsRIP18 = RA39	Tapetum	Not reported	Involved in drought and salt response. Involved in microspore maturation?
	Other rice RIPs	Variable	Expression of several genes induced by *Magnaporthe grisea* or *Xanthomonas oryzae*	Expression of several genes is enhanced after abiotic stress
*Triticum aestivum*	Tritin	Seed and leaf forms	Not reported	Involved in senescence
*Hordeum vulgare*	RIP30 and isoforms	Seeds	Antifungal protein	Not reported
	JIP60	Leaves	Re-organization of translational machinery in stress situations	Re-organization of translational machinery in stress situations
